# Supplementary material for: Computational Assessment of Spectral Heterogeneity within Fresh Glioblastoma Tissue Using Raman Spectroscopy and Machine Learning Algorithms
Source: Molecules. 2024 Feb 23;29(5):979. doi: 10.3390/molecules29050979 (PMC10935394; doi:10.3390/molecules29050979)
Supplement: Supplementary file 1 [file molecules-29-00979-s001.zip › molecules-2743982-supplementary.pdf]

Supplemental Information

Data preparation

Initially data were split independently in a training and validation cohort (approx. 3:1) in which the number of measurements per patient was randomly reduced to 15. Both reduce the impact of potential patient specific classification and inherent data imbalance and allow generalization over each class. Subsequent machine learning algorithms and display of results were employed in Python using standard libraries such as scikit-learn, plotly and matplotlib.

Binary Random Forest classification, UMAP and K-means clustering

Best model showed: max depth of the trees [10], minimum sample to consider a leaf [2], minimum samples to consider a split [5]. Employing the best decision threshold (0.452 - based on maximized f-1 score) of the previous internal CV, data re-curation and separation of data in necrotic and non-necrotic spectral data was conducted.

UMAP (Uniform Manifold Approximation and Projection) was performed and established with n_neighbors=[20] and min_dists=[0.01].

After the following k-means clustering analysis, k-means clusters (best n=21) were visualized within the spatial distribution of the previous UMAP and cluster assignments of measurements from a healthy autopsy brain as well as measurements of infiltration zones were predicted.

Supplemental Material – Figures

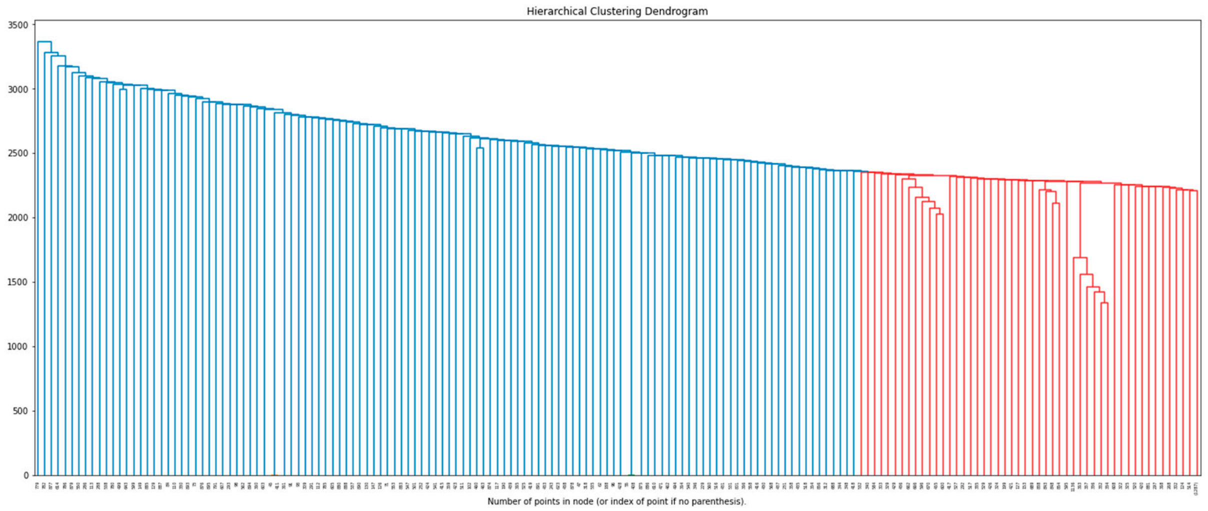

Supp. Figure S1. To initially determine strong artifacts and outliers, a distance-based hierarchical dendrogram was displayed.

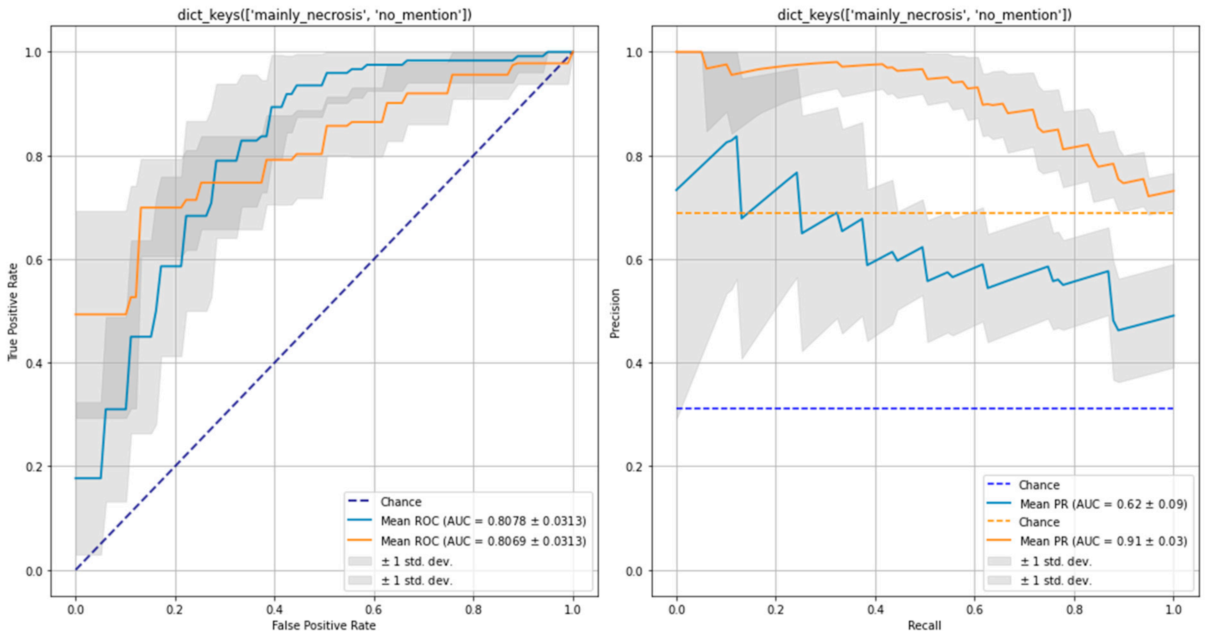

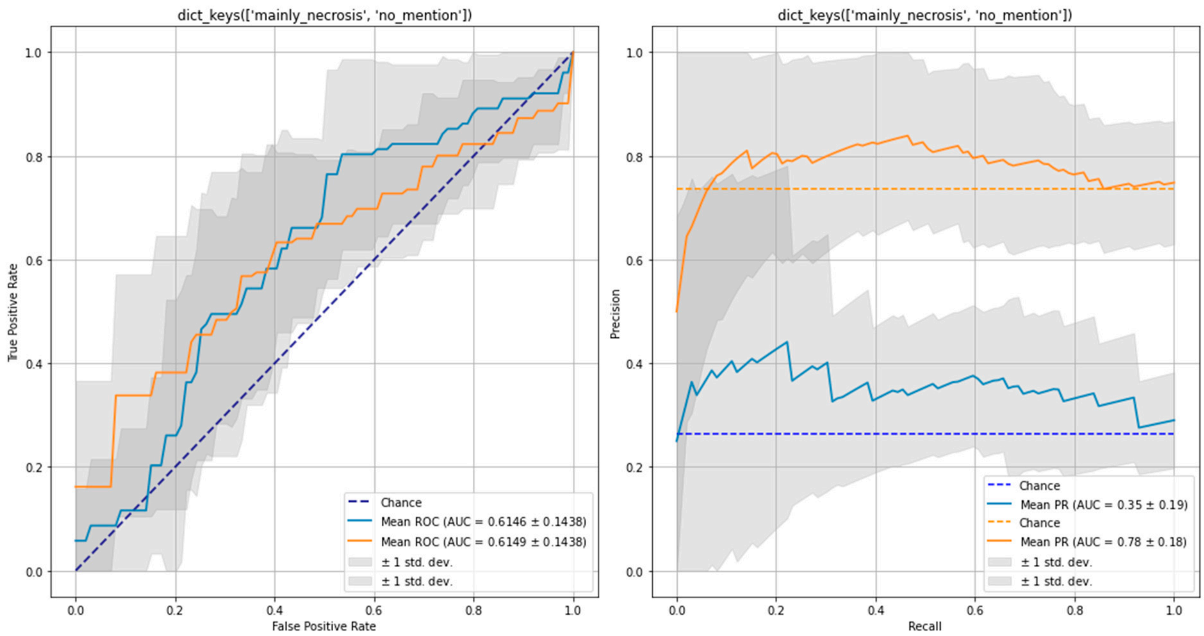

Supp. Figure S2. Evaluation of classifier performance using the external data set. Above: Cross validation with random data distribution. Below: Cross validation with patient-dependent data distribution.

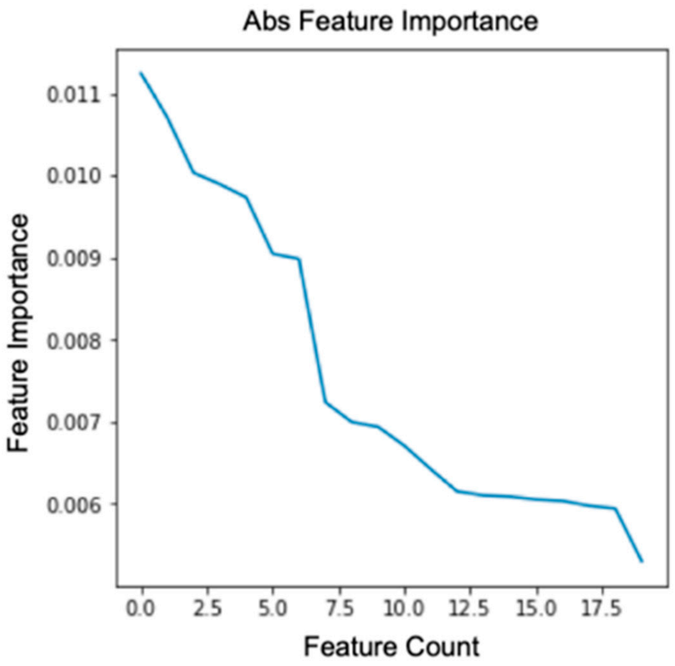

Supp. Figure S3. The feature importance analysis indicated a high complexity within the spectroscopic data since relevant features cannot be limited to a few frequency bins.

A

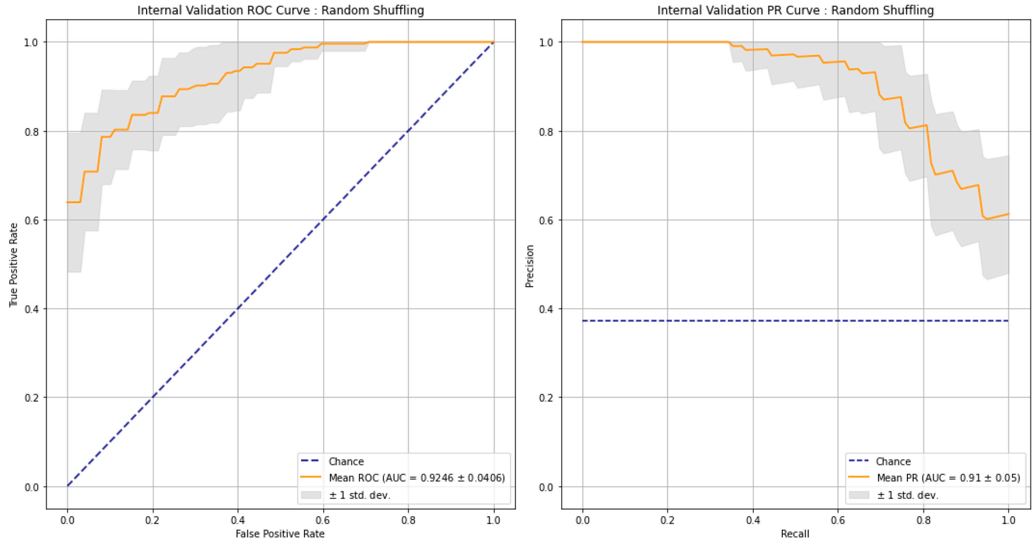

B

```
Best threshold (max gmean score) : 0.452
Best mean gmean score : 0.8456352282912019
Corresponding std at argmax best gmean score : 0.058781303188335084

Sensitivity = 0.7772058823529411 +- 0.09958463331567888
Specificity = 0.9239858906525573 +- 0.03444530685215861
Precision = 0.8604442401423827 +- 0.054085708439185066
Accuracy = 0.8694855532064835 +- 0.045240357193596425
Balanced accuracy = 0.8505958865027494 +- 0.05467495168594882
AUROC = 0.924349647266314 +- 0.04056419901593744
AUPR = 0.9051073004272905 +- 0.046841048369691295
```

Supp. Figure S4. A: ROC and PR curve of the internal cross validation of our Random Forest classification. B: Corresponding metrices.

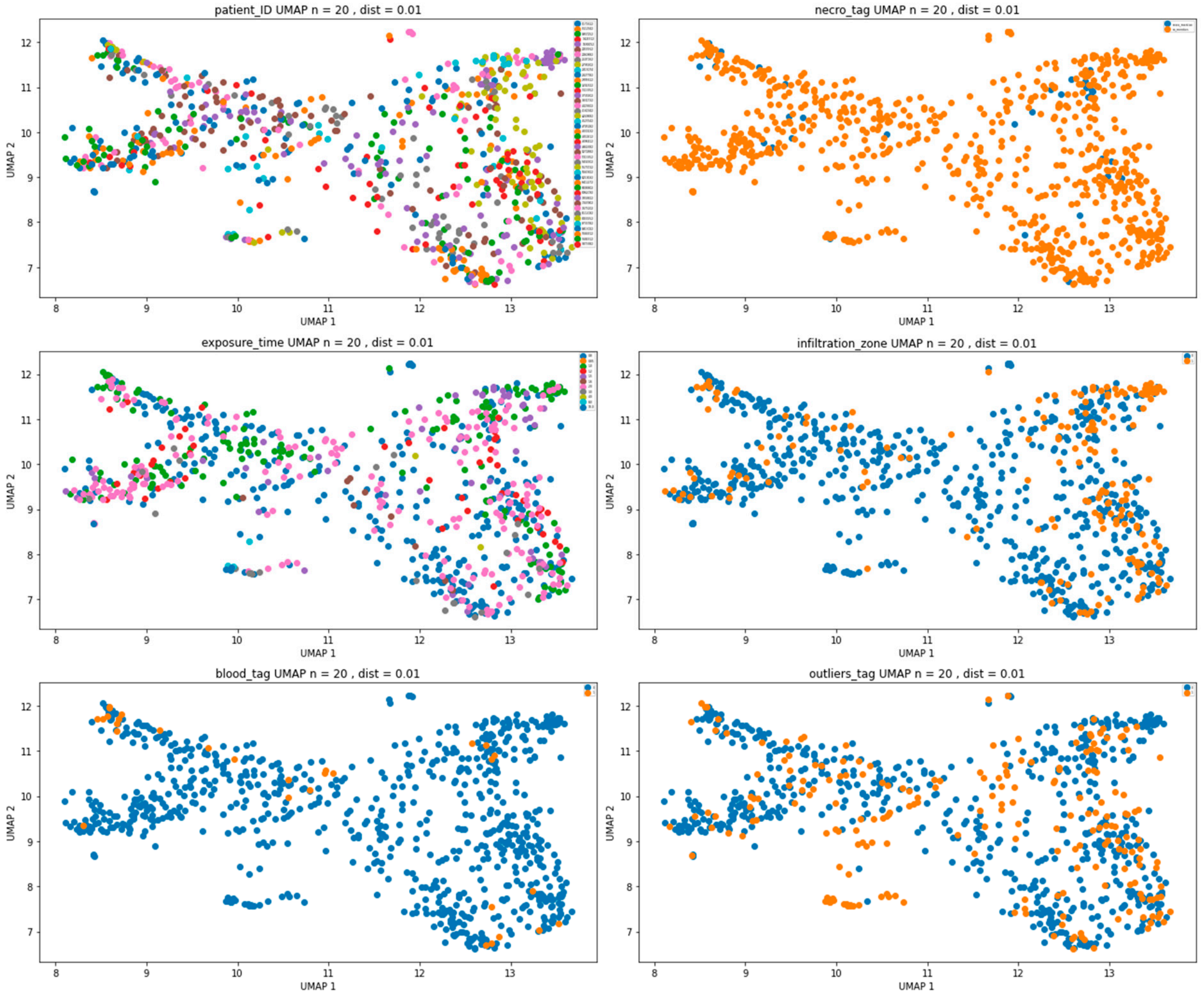

Supp. Figure S5. UMAP displacement reveals no distinct technical confounders (patient IDs and different exposure times) or biological differences (infiltration zone and hemorrhage).

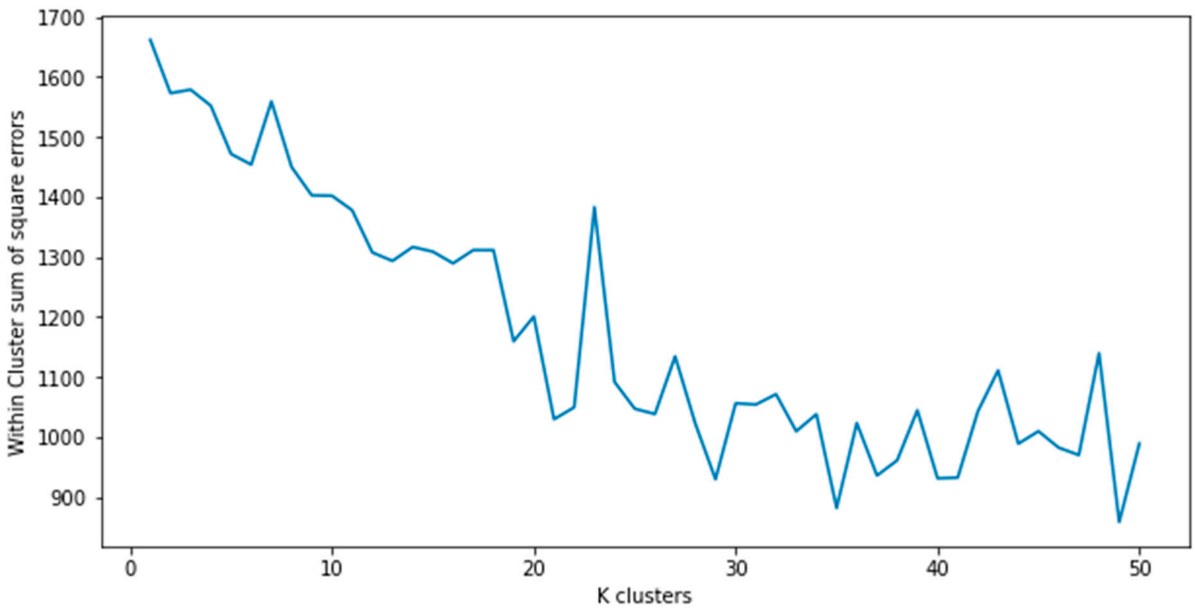

Supp. Figure S6. Evaluation of best k within kmax=50 on spectrally non-necrotic tissue.

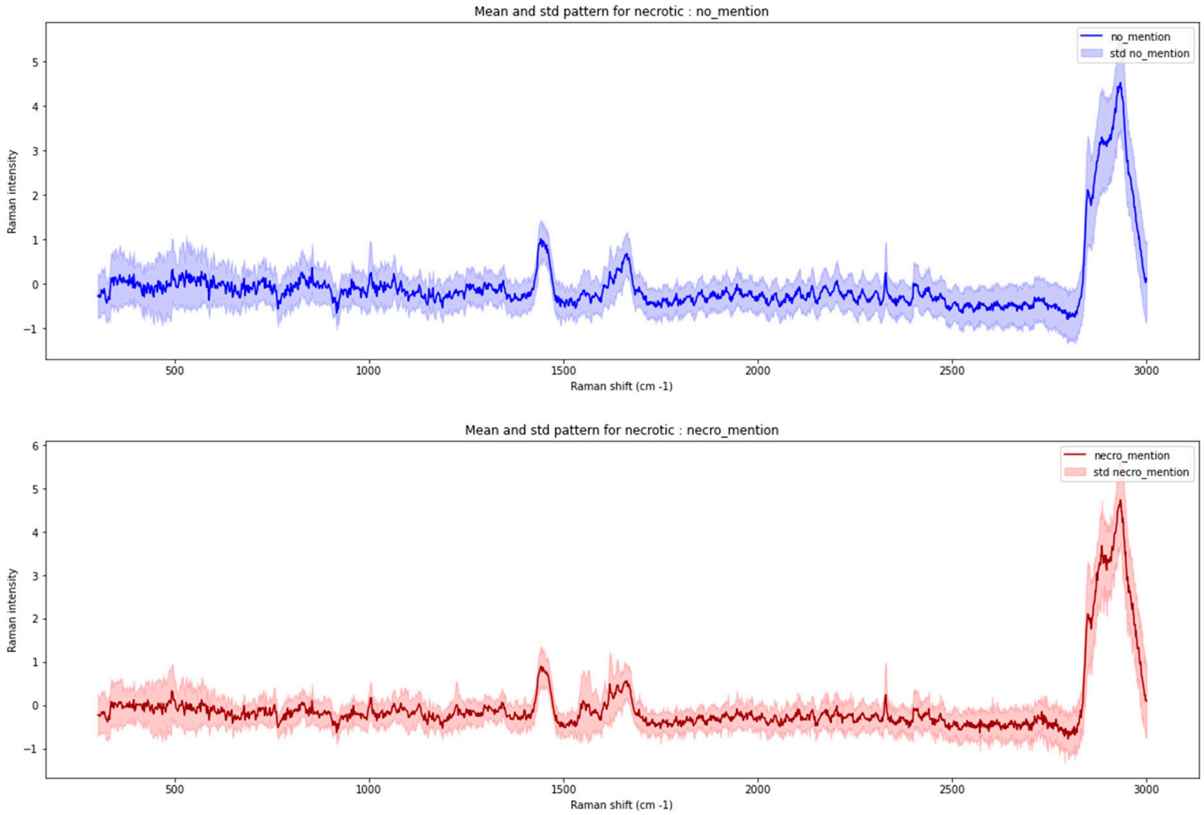

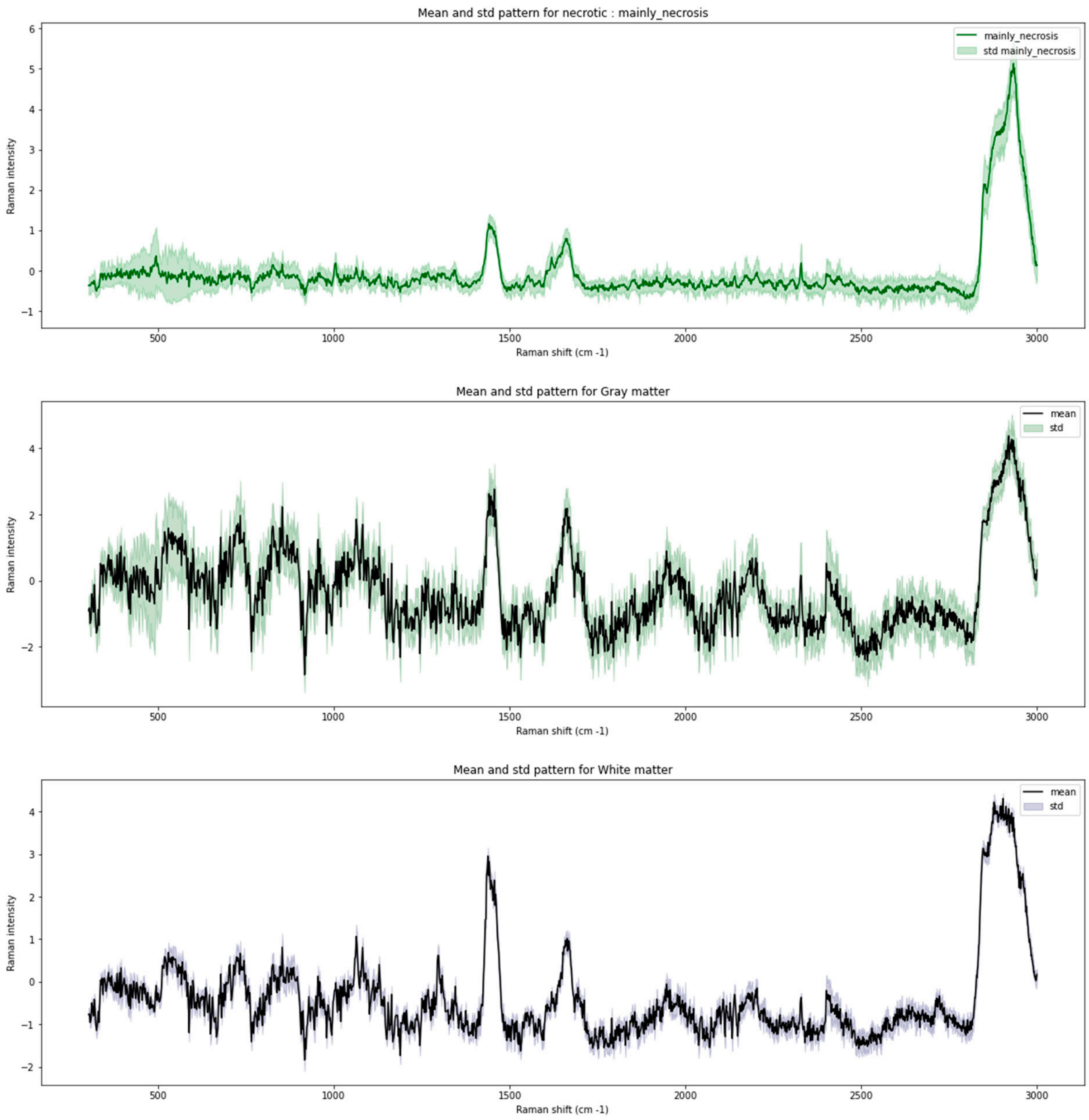

Supp. Figure S7. Data display. Mean and standard deviation for all three data classes ((‘*necrosis data set*’, ‘*vital data set’, heterogenous data set’*) and autopsy brain tissue (‘*grey matter’, ‘white matter*’).

Supplemental Material – Tables

patient no°	age	sex	localization	IDH status	MGMT status	primary / secondary / recurrence tumor
1	72	female	right temporal	wildtype	methylated	primary
2	72	male	right opercular	wildtype	methylated	primary
3	64	male	right fronto-dorsal	wildtype	methylated	primary
4	50	female	left temporal	inconclusive: highest score wildtype	methylated	primary
5	64	female	right occipital	wildtype	methylated	primary
6	49	male	left fronto-temporal	wildtype	methylated	primary
7	58	male	supratentorial	wildtype	un-methylated	primary
8	27	male	left frontal	mutant	un-methylated	not specified
9	75	female	right central	wildtype	un-methylated	secondary & recurrence
10	67	male	left frontobasal & left frontal	wildtype	un-methylated	primary & recurrence
11	50	female	bifrontal	wildtype	methylated	primary
12	64	male	right temporal	wildtype	methylated	primary

13	66	male	two tumors, left frontal	wildtype	methylated	primary
14	70	male	left temporo-dorsal	wildtype	un-methylated	primary
15	66	male	left temporal	wildtype	un-methylated	primary
16	64	female	right parietal	wildtype	un-methylated	recurrence
17	41	female	right temporal	mutant	inconclusive	secondary
18	62	male	left temporal	wildtype	un-methylated	primary
19	45	female	right temporal	inconclusive: highest score wildtype	un-methylated	recurrence
20	61	m	right parieto-occipital	wildtype	methylated	primary
21	61	male	right temporal	wildtype	methylated	recurrence
22	53	male	corpus callosum	wildtype	methylated	primary
23	57	male	right frontal	wildtype	un-methylated	primary
24	55	female	left occipital	wildtype	un-methylated	recurrence
25	75	female	right parietal	wildtype	un-methylated	recurrence

26	67	male	temporo-insular	wildtype	not specified	primary
27	80	male	left frontal	wildtype	methylated	Propably secondary
28	56	male	left temporo-occipital	inconclusive: highest score wildtype	un-methylated	3rd recurrent tumor
29	81	male	left temporal	wildtype	methylated	primary
30	62	male	right occipital	wildtype	methylated	rest / recurrence
31	44	female	right frontal	wildtype	un-methylated	primary
32	69	male	left fronto-parietal	wildtype	un-methylated	primary
33	44	male	temporo-mesial	wildtype	un-methylated	recurrence
34	47	male	left marginal gyrus & parietal lobe	wildtype	methylated	primary
35	69	female	multifocal	wildtype	methylated	primary
36	48	female	not specified	wildtype	un-methylated	primary
37	64	male	left temporal & parietal	wildtype	methylated	primary
38	61	male	frontoparietal	wildtype	methylated	primary
39	66	male	parietal	wildtype	not specified	primary

40	37	male	right temporal	wildtype	not specified	primary & recurrence
41	50	male	right temporal	wildtype	un-methylated	primary
42	67	male	right parietal	wildtype	methylated	recurrence
43	58	male	left occipital	wildtype	un-methylated	primary & recurrence

Supp. Table S1 lists all tumors included with corresponding patient's characteristics (age, sex), as well as clinical and biological features (localization, IDH-status (brain tumor classification classifier results (v11b4): wildtype/mutant; results with matching score <0.9 were considered inconclusive), MGMT-status (unmethylated/methylated), tumor recurrence).
